# Human-facility interaction improving people’s understanding of service robots and elevators - system design and evaluation

**DOI:** 10.3389/frobt.2025.1681187

**Published:** 2025-11-03

**Authors:** Mau Adachi, Masayuki Kakio

**Affiliations:** Advanced Technology R&D Center, Mitsubishi Electric Corporation, Hyogo, Japan

**Keywords:** smart elevator, social elevator, social robot, human-robot interaction, human-elevator interaction

## Abstract

As service robots become increasingly integrated into public spaces, effective communication between robots and humans is essential. Elevators, being common shared spaces, present unique challenges and opportunities for such interactions. In this study, we developed a Human-Facility Interaction (HFI) system to facilitate communication between service robots and passengers in elevator environments. The system provided both verbal (voice announcements) and non-verbal (light signals) information to passengers waiting for an elevator alongside a service robot. We installed the system in a hotel and conducted two experiments involving 31 participants to evaluate its impact on passengers’ impressions of the elevator and the robot. Our findings revealed that voice-based information significantly improved passengers’ impressions and reduced perceived waiting time. However, light-based information had minimal impact on impressions and unexpectedly increased perceived waiting time. These results offer valuable insights for designing future HFI systems to support the integration of service robots in buildings.

## 1 Introduction

In recent years, service robots, such as delivery robots and security robots, have increasingly gained the capability to use elevators, enabling them to provide services across multiple floors of buildings (López, et al., 2013; Collin, et al., 2023; Palacín, et al., 2023; Al-Kodmany, 2023; Panasonic, 2015). Many studies have focused on the technological functions that enable robots to use elevators, such as the identification of the control panels (Klingbeil, et al., 2010; Yu, et al., 2019; Zhu, et al., 2020; Zhu, et al., 2021) and their operation (Ali, et al., 2017; Liebner, et al., 2019; Zhu, et al., 2020). Another approach is to enhance elevators, enabling direct communication between elevators and service robots (López, et al., 2013; Panasonic, 2015; Abdulla, et al., 2017; Robal, et al., 2022). Currently, several elevator companies have also developed systems known as “smart elevators” to assist robots in moving between multiple floors within buildings (Mitsubishi Electric Building Solutions Corporation, n.d.; KONE Corporation, n.d.; OTIS, 2024). Smart elevator systems allow service robots to call an elevator to their current floor, board it, and travel to their desired destination.

With the increasing use of elevators by service robots, there is a growing need for service robots and humans to share the same elevator to improve transportation efficiency. Consequently, it has become more important to inform surrounding passengers when a robot is using the elevator. Some studies have begun to examine more socially acceptable behaviors of robots when sharing an elevator with passengers. This includes communication methods to notify passengers of robot boarding (Babel, et al., 2022; Law, 2022), waiting position design inside and outside an elevator (Gallo, et al., 2022), and trajectory design for entering an elevator (Gallo, et al., 2023; Kim, et al., 2024). Unfortunately, current robots lack their computational resources needed to achieve advanced social behaviors, and they do not yet have fully developed interfaces for conveying their intentions. It will thus take a long time before all service robots deployed in buildings possess such capabilities. On the other hand, facilities within buildings, such as elevators, often already have some methods for interacting with users, such as speakers. Therefore, having facilities interact with users instead of robots should be a beneficial strategy. However, the impact of social interactive behaviors by facilities on the social acceptance of both robots and the facilities themselves has been scarcely examined.

In the pursuit of a society where humans and robots collaborate, numerous studies have explored robots as subjects of human interaction, emphasizing the understanding and emotional responses that humans exhibit toward robots, as well as the robot behavior designs within the context of Human-Robot Interaction (HRI) (Fong, et al., 2003; Breazeal, 2004; Rodríguez-Guerra, et al., 2021; Stock-Homburg, 2022). On the other hand, we have focused on the social behaviors of elevators as ‘autonomous agents.’ In our previous studies (Shiomi, et al., 2024; Shiomi, et al., 2025), we examined how the design of voice cues provided by a robot and/or an elevator affects passengers’ impressions when the robot takes the elevator. We then found that passengers’ impressions of both the robot and the elevator can improve when at least one of them, either the robot or the elevator, speaks. Based on these findings, we developed the concept that high-function facilities in buildings can facilitate smooth interactions between humans and service robots by supporting the social behaviors of the robots. We refer to this concept as “Human-Facility Interaction (HFI),” inspired by the term human-robot interaction.

In the field of robotics, many studies have investigated various verbal (e.g., speech and text display by robots) and non-verbal communication expressions (e.g., gestures and lighting from robots) for social robots that incorporate interfaces for communicating with people (Kanda, et al., 2002; Imai, et al., 2003; Breazeal, 2003; Breazeal, 2003; Breazeal, 2004; Bethel and Murphy, 2008; Marin Vargas, et al., 2021). However, unlike robots, facilities in buildings lack clear embodiments, limiting the ways they can communicate with users. For facilities in buildings, one possible way to communicate with users is using voice announcements, which are commonly used in elevators. When essential information is summarized in short sentences concisely, voice announcements can effectively convey accurate information. However, voice announcements are limited by language barriers and cannot reach non-native speakers or individuals with hearing impairments. Therefore, to accommodate a diverse range of users in the future, HFI systems will need to incorporate non-verbal communication methods as well.

In this study, we focused on the scenario in which a robot boards an elevator and developed an HFI system that provides verbal and non-verbal information to users. Specifically, the system offered voice and light-based information to passengers waiting for the next elevator in an elevator hall with a service robot, explaining the status of the elevator and the robot. We then installed it in a hotel to conduct demonstrations and investigated the effects of information on passengers’ impressions of the elevator and the robot.

## 2 System design

### 2.1 Concepts

We focused on a scenario where a service robot boards an elevator with a passenger. To prevent collisions between service robots and passengers, the boarding timings for both should be properly defined and clearly separated. We thus defined the following phases for the scenario:

Phase 0: no service robot is waiting for the elevator in the elevator hall.

Phase 1: while a passenger is waiting for the elevator car, a service robot arrives at the elevator hall to board.

Phase 2: when the elevator arrives and the doors open, the passenger gets on the elevator before the service robot.

Phase 3: after the passenger has boarded, the robot enters the elevator car, both the robot and the passenger wait for the elevator to depart, and other passengers in the elevator hall stay clear from the elevator.

**FIGURE 1:**
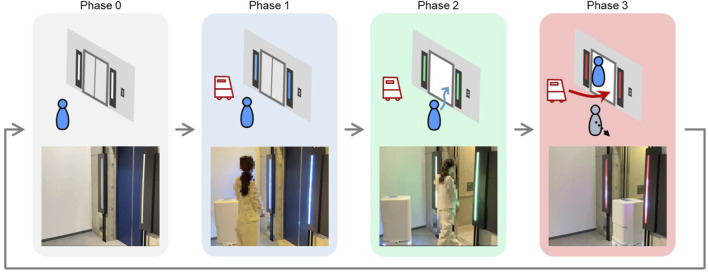
System concept and actual system operation.

Figure 1 illustrates our concept and the phases above. When the elevator departs in Phase 3, Phase 3 ends and Phase 0 starts again. Since passengers can move faster than service robots, we defined the phases so that the passenger enters the elevator car first, followed by the service robot.

### 2.2 Designed contents

Along each phase described in the previous section, we designed voice announcement contents accordingly (Table 1). In Phase 1, the announcement informs passengers at the elevator hall that the service robot will board the next elevator and asks them to enter the elevator before the robot starts to move. In Phase 2, the announcement briefly encourages the passengers to board before the robot. In Phase 3, the announcement explains that the robot is starting to board the elevator, tells the passenger inside the elevator car to wait for a while, and urges potential passengers still in the elevator hall not to enter for safety reasons. We designed short notification sounds and added them to the beginnings of the announcements in Phases 2 and 3, so that people could easily recognize phase changes using only audio information.

**TABLE 1:** Announcements for each phase.

Phase	Announcements	
	English translations	Original sentences in Japanese
1	- The elevator is shared with the robot. It will also take the next elevator. Please board the next elevator before the robot starts boarding. - The robot is waiting for the next elevator. The robot will board after you. Please board first and leave a wide space in the center of the elevator.	- こちらはロボットと共用のエレベーターです. 次のエレベーターにロボットが乗車します. お客様はお先に乗車してください. - ロボットはエレベーターを待っています. お客様に続いてロボットが乗車します. お先にご乗車いただき中央付近を広く空けて お待ちください.
2	- This is an elevator shared with robots. Please board the elevator now.	- こちらはロボットと共用のエレベーターです. お客様はお先にどうぞ.
3	- The robot starts boarding. Please leave enough space near the doors. - The elevator is now checking for safety. Please wait away from the robot for a while. - For your safety, please refrain from boarding the elevator now and watch the closing doors.	- ロボットが乗車します.扉付近を広く空けてお待ちください. - 安全の確認を行っています.ロボットから離れて今しばらくお待ち ください. - 安全のためこれからのご乗車はお控えいただき,閉まる扉にご注意 ください.

For the light-based information, we designed a lighting color for each phase based on traffic signals. In Phase 1, the light units emit blue light to calm the passengers and reduce their stress (Gorn, et al., 1997; Gorn, et al., 2004; Valdez and Mehrabian, 1994). In Phase 2, the light units emit green light to encourage the passenger to board the elevator, similar to a traffic light. In Phase 3, the light units emit red light to prohibit additional passenger boardings. We also designed wavy lighting patterns to give passengers the impression that the system was processing.

### 2.3 Developed system

**FIGURE 2:**
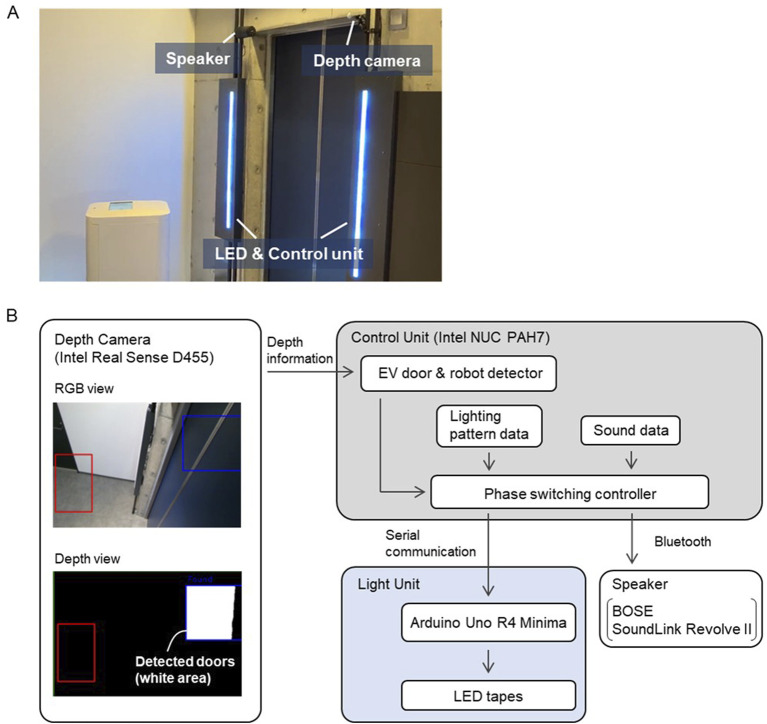
Developed system. **(A)** Installation in elevator hall. **(B)** System diagram.

Figure 2 shows the developed HFI system. We installed it on both sides of the elevator doors. The system had two interaction methods: a speaker for voice announcements (BOSE SoundLink Revolve II) and two light units. The speaker and light units could each be turned on or off by the operator. To detect the arrival of a service robot and the elevator car using depth information, our system had a depth camera (RealSense D455) near the top of the elevator doors. The specific detection strategy was as follows. First, the depth camera was adjusted so that both a robot waiting at a specified location in the elevator hall and the elevator doors were within its field of view. The operator then designated rectangular areas for the robot and the elevator doors in the RGB image obtained in real time by the depth camera, which included the robot or the doors, respectively. We detected the arrival of the robot or the opening and closing of the elevator doors based on changes in the depth information of the point cloud obtained within those rectangular areas.

The system operates according to each phase described in Section 2.4. When the current state is Phase 0, no voice announcement or light is provided. When the depth camera detects the robot’s arrival in Phase 0, the control unit switches the current state to Phase 1 and starts to play the voice announcements and lighting patterns for Phase 1 repeatedly. When the depth camera detects the elevator doors opening in Phase 1, the control unit then switches the current state to Phase 2 and plays the corresponding voice announcement and lighting patterns. When a certain period elapses in Phase 2, the control unit automatically switches the current state to Phase 3 and plays the corresponding voice announcement and lighting patterns. When another certain period elapses, the control unit automatically switches the current state to Phase 0 and stops the voice announcement and lighting patterns.

For passengers who are unfamiliar with boarding an elevator with a service robot, it is important to provide information at the appropriate time (Bacotti, et al., 2021). To ensure that the timing of announcements and lights matched the actual events, we determined the durations of Phases 2 and 3 based on the actual boarding time of the service robot used in the experiments.

## 3 Experiments

### 3.1 Hypothesis

Our system can present information by light as well as by sound. As explained earlier, our previous studies have shown that voice announcements improve passengers’ impressions of both the elevator and the robot (Shiomi, et al., 2024; Shiomi, et al., 2025). In addition, light-based information will help users in an elevator hall to understand the status of the elevator and the boarding behavior of service robots. It is generally expected that providing more feedback methods will reduce user’s perceived waiting time (Branaghan and Sanchez, 2009) and also decrease their stress while waiting (Osuna, 1985; Bird, et al., 2016; Fan, et al., 2016). We thus formulated the following hypothesis.

H1 When the system provides more information, participants will have more positive impressions of both the elevator and the robot.

H2 When the system provides more information, participants will perceive a shorter waiting time for the elevator with the robot to depart.

H3 When the system provides different colors with its light units, participants will be able to decide whether to board the elevator with the robot.

We evaluated H1 and H2 in Experiment A and H3 in Experiment B.

### 3.2 Participants

Thirty-one people participated in the experiments: 16 women and 15 men. Their ages ranged from their 20s to 60s, with an average age was 41.0 (S. D. = 11.5). They were recruited through a temporary employment agency and received monetary compensation for their participation. They received 5,000 yen per hour as compensation for participating in the experiments.

### 3.3 Environment

**FIGURE 3:**
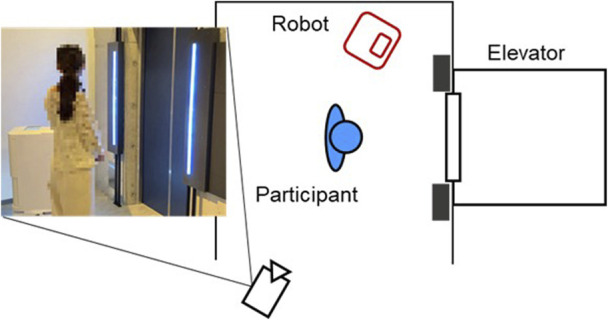
Experimental environment.

Figure 3 illustrates the experimental environment. We conducted the experiments in Tap Hospitality Lab Okinawa (THL). THL is a demonstration accommodation facility in Japan where regular tourists can also stay. We used an elevator in THL with an interior size of approximately 2.3 m in height, 1.7 m in width, and 1.7 m in depth, which incorporates a system that enables robots to move freely between floors (Mitsubishi Electric Building Solutions Corporation, n.d.). We used a delivery robot (YUNJI GOGO: 0.98 m tall, 0.42 m wide, and 0.49 m deep) (YUNJI TECHNOLOGY, n.d.). The robot had a white cuboid shape and featured an operation panel on its top surface as an interface. It also possessed the ability to autonomously navigate to user-specified destinations while avoiding obstacles and collaborating with the elevator. The robot was set up to travel back and forth between two locations on different floors in the building.

### 3.4 Conditions

**FIGURE 4:**
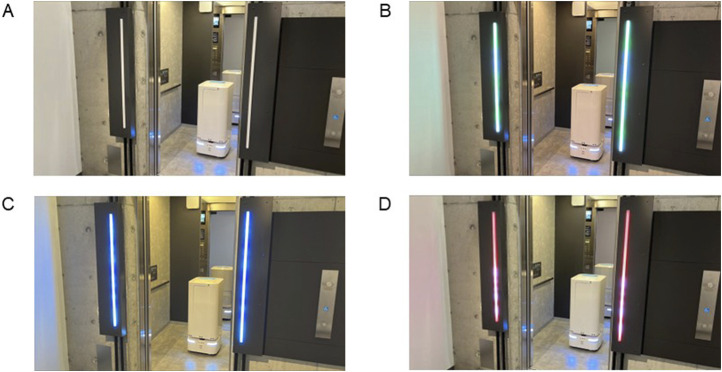
Robot and elevator with system’s light units in different states. **(A)** No lights. **(B)** Green lights. **(C)** Blue lights. **(D)** Red lights.

For Experiment A, we considered two factors: *sound* (*with sound* or *without sound*) and *light* (*with light* or *without light*). For Experiment B, we considered one factor: the state of the light units (no lights, green lights, blue lights, or red lights, see Figure 4). We thus prepared four conditions for each experiment.

### 3.5 Measurements

For Experiment A, we evaluated the perceived impressions of both the elevator and the robot using existing questionnaire scales (Bartneck, et al., 2009): likability, intelligence, and safety. Each item was rated on a 7-point scale, with 1 indicating the least favorable response and 7 the most favorable. We also asked participants to evaluate their perception of the waiting time it took for the elevator doors to close, compared to the typical duration we had measured in advance. For Experiment B, we evaluated the degree of hesitation when boarding the elevators shown in Figure 4 using a 1–9 response format, with 1 indicating the least hesitation and 9 the most hesitation.

### 3.6 Procedure

All the procedures were approved by the Ethics Review Committee of Advanced Technology R&D Center (ATC 2024-002). First, the participants read explanations of the experiments and how to evaluate the service robot and the elevator in each condition. We employed a within-participant design in which the participants experienced four different conditions in Experiment A. We first conducted Experiment A followed by Experiment B without explaining the hypotheses to the participants. After starting Experiment A, the participants first waited for the elevator in the hall, and then the robot arrived. When the elevator car arrived, the participants boarded it and waited for the robot to board and the elevator to depart. After the elevator arrived at the destination floor, the participants exited the elevator and answered questionnaires. Before each trial, we moved the elevator to a different floor to allow the participants to experience the waiting time for the elevator to arrive at the hall. We measured the time between the elevator door opening and closing for each trial to normalize participants’ subjective waiting time using the objective duration. The order of the conditions was counterbalanced. After Experiment A, the participants were shown a figure similar to Figure 4 and completed questionnaires for Experiment B. At the end of the experiments, we conducted a brief interview with the participants.

## 4 Results

### 4.1 Impressions of system and perceived waiting time at elevator boarding

**FIGURE 5:**
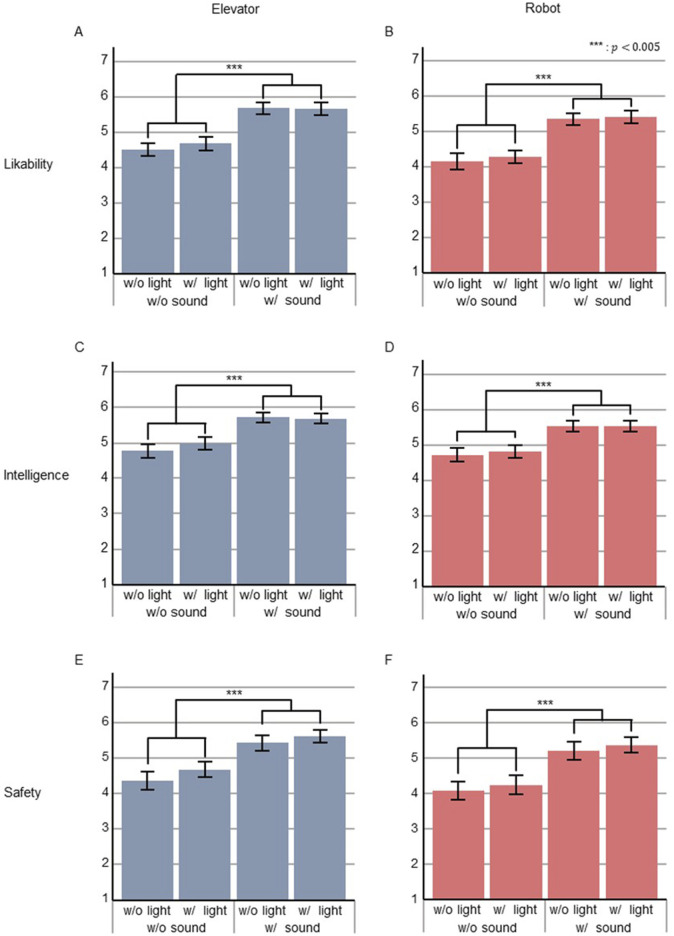
Questionnaire results of perceived likability **(A,B)**, intelligence **(C,D)**, and safety **(E,F)** of elevator (left) and robot (right).

We conducted a two-way factorial (*sound* and *light*) ANOVA to analyze the questionnaire results regarding the impression scales of Experiment A (Figure 5). The statistical analysis of the elevator’s likability scale showed a significant difference in the *sound* factor ( $F\left(1,30\right)=54.3,p=3.32\times 10^{-8},partial\eta^{2}=0.64$ ), and no significant differences in the *light* factor or in the interaction effects (Figure 5A). The statistical analysis of the robot’s likability scale showed a significant effect of the *sound* factor ( $F\left(1,30\right)=47.7,p=1.14\times 10^{-7},partial\eta^{2}=0.61$ ), and no significant differences in the *light* factor or in the interaction effects (Figure 5B). The statistical analysis of the elevator’s intelligence scale showed a significant difference in the *sound* factor ( $F\left(1,30\right)=35.8,p=1.47\times 10^{-6},partial\eta^{2}=0.54$ ), and no significant differences in the *light* factor or in the interaction effects (Figure 5C). The statistical analysis of the robot’s intelligence scale showed a significant effect of the *sound* factor ( $F\left(1,30\right)=23.7,p=3.41\times 10^{-5},partial\eta^{2}=0.44$ ), and no significant differences in the *light* factor or in the interaction effects (Figure 5D). The statistical analysis of the elevator’s safety scale showed a significant effect of the *sound* factor ( $F\left(1,30\right)=31.0,p=4.66\times 10^{-6},partial\eta^{2}=0.51$ ), and no significant differences in the *light* factor or in the interaction effects (Figure 5E). Finally, the statistical analysis of the robot’s safety scale showed a significant effect of the *sound* factor ( $F\left(1,30\right)=36.0,p=1.40\times 10^{-6},partial\eta^{2}=0.55$ ), and no significant differences in the *light* factor or in the interaction effects (Figure 5F).

**FIGURE 6:**
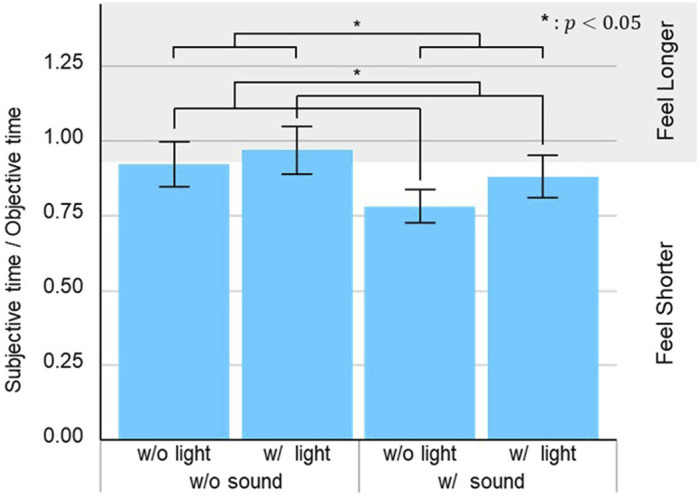
Questionnaire results for perceived waiting time ratio. Grey region indicates area where participants perceived the waiting time was longer compared to the *without light or sound* condition, while white region indicates area where participants perceived the waiting time was shorter.

Regarding the waiting time results of Experiment A, we first normalized subjective waiting time by dividing it by the corresponding objective waiting time for each trial. Throughout all trials, the elevator took an average of 42.5 s to close its doors after opening, with a standard deviation of 2.7 s. We then conducted a two-way factorial (*sound* and *light*) ANOVA to analyze the waiting time results (Figure 6). The statistical analysis showed a significant effect of the *sound* factor ( $F\left(1,30\right)=5.06,\;p=0.032,\;partial\eta^{2}=0.14$ ) and in the *light* factor ( $F\left(1,30\right)=6.10,\;p=0.019,\;partial\eta^{2}=0.17$ ), and no significant differences in the interaction effects.

### 4.2 Impressions of system by passengers in elevator hall

**FIGURE 7:**
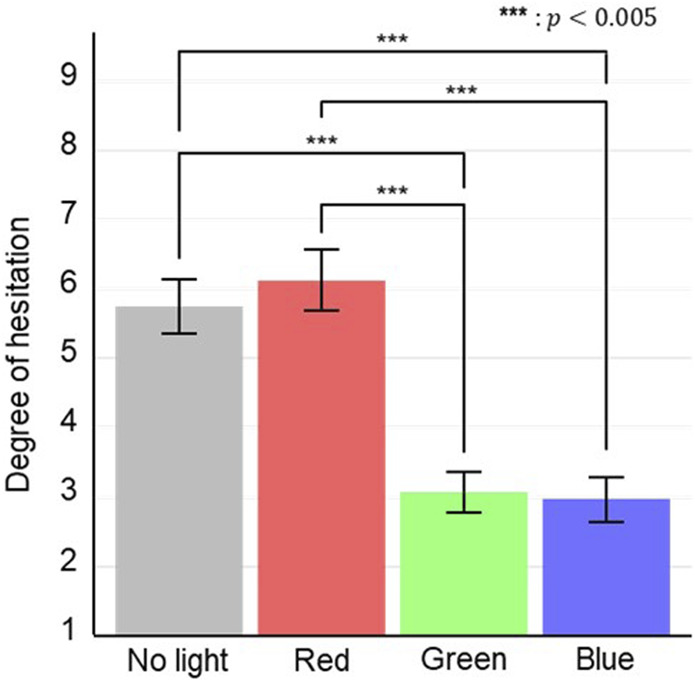
Questionnaire results of degree of hesitation for states of light unit.

We conducted a one-way factor ANOVA to analyze the questionnaire results regarding the impression scale of Experiment B (Figure 7). The statistical analysis of the degree of hesitation scale showed a significant effect of the light unit state factor ( $F\left(3,90\right)=22.3,\;p=7.07\times 10^{-11},partical\eta^{2}=0.43$ ). A Bonferroni-corrected pairwise comparison as a *post hoc* test showed significant differences: *green lights* < *no lights* ( $t\left(90\right)=5.28,p=5.40\times 10^{-6},Cohen^{\prime }s\;d=1.40$ ), *green lights* < *red lights* ( $t\left(90\right)=6.04,$ $p=2.04\times 10^{-7},Cohen^{\prime }s\;d=1.48$ ), *blue lights* < *no lights* ( $t\left(90\right)=5.47,p=2.42\times 10^{-6},Cohen^{\prime }s\;d=1.38$ ), and *blue lights* < *red lights* ( $t\left(90\right)=6.23,p=8.76\times 10^{-8},Cohen^{\prime }s\;d=1.46$ ).

Most participants mentioned the color of the light units as the reason for their responses ( n=26 ). Sixteen of the participants gave clearly negative reactions to the *red lights* condition, of whom thirteen indicated that red signifies danger or prohibition, and six mentioned its similarity to traffic signals. On the other hand, only four of the participants gave clearly negative reactions to the *no lights* condition. Their reasons also varied, such as: “With no lights, it was difficult to determine whether the robot was getting on or off,” and “No lights lack distinctive colors, making it hard to understand what signal is being conveyed.” Nineteen of the participants gave clearly positive reactions to the *green lights* or *blue lights* condition, of whom five indicated its similarity to traffic lights. Other comments included, “Green lights seemed the gentlest,” and “Blue lights were the most relaxing.”

## 5 Discussion

Our results showed that providing voice-based information improved passengers’ impressions of the elevator and the robot when they boarded with the robot in a statistically significant manner (Figure 5). This is consistent with the results of our previous studies (Shiomi, et al., 2024; Shiomi, et al., 2025). In those studies, we also found an implication that when two agents are present and one of them speaks, it may be sufficient to change the perceived attributes of the other. In this study, we did not include information about the identity of the guide in the announcements. In post-experiment interviews, some participants said that the elevator talked, while others believed it was the robot. This would correlate with our earlier implication. On the other hand, although there were some differences in the mean values of each impression item, our results showed that providing light-based information statically had little effect on passengers’ impressions of the elevator and the robot (Figure 5). In post-experiment interviews, some participants reported that they did not notice the lights at all. However, about one-third of participants ( n=9 ) reported that the system with both voice-based and light-based information was the most favorable. This suggests that providing light-based information would reinforce the effects of voice-based information and help passengers understand the status of the elevator and robot. Based on these findings, H1 was partly supported.

Waiting time for a service to be provided has a significant impact on the user’s stress when that service is delivered (Pruyn and Smidts, 1998; Bielen and Demoulin, 2007; Ayodeji, et al., 2023). Our results showed that providing voice-based information reduced the perceived waiting time between elevator arrival and departure in a statistically significant manner. On the other hand, statistical analysis also showed that providing light-based information significantly increased the perceived waiting time (Figure 6). H2 was therefore unsupported. That is contrary not only to our expectations, but also to the findings of previous studies (Bartneck, et al., 2009). In general, multiple forms of feedback will contribute to a greater reduction in perceived waiting time. Our experiment could have caused this discrepancy for several reasons. One possible reason is that the participants could not see the light units once they were in the elevator car. When the light units were activated and the participants were waiting in the elevator hall, the participants could see the blue lighting patterns designed for Phase 1. If participants saw blue lights, they likely felt calmer and perceived the elevator arrival time as shorter (Gorn, et al., 1997; Gorn, et al., 2004; Valdez and Mehrabian, 1994). However, once they entered the elevator, they could no longer see the color of the light units from inside. As a result, they possibly felt the time until the elevator departed was longer, in contrast to before they boarded the elevator. Another possible reason is that the light-based information provided was unclear to the participants who were seeing it for the first time. When participants felt that the lights were unclear or incomplete, the use of light-based information may have inadvertently increased the cognitive load on them, contrary to our intention. Regarding the robot’s interface, some previous studies have reported that when users experience a robot’s behavior with light-based information, they can correctly understand the function of the lights (Fernandez, et al., 2018). Even if designers meticulously create an interface using lights, users still need to become familiar with the light-based information from a new device to utilize it for a quick understanding of the situation.

Our results also showed that the color of the system lighting significantly affects the degree of hesitation passengers feel about entering the elevator in the hall (Figure 7). Most participants also had a good understanding of the color, even though we did not explain it before the experiments. Therefore, H3 was supported. Even though the color of the system lighting did not contain specific information, cultural context may have allowed participants to infer its meaning. To clarify the information conveyed by the lights and enhance their effectiveness in Phase 3, it would be beneficial to use a lighting representation similar to countdown displays on traffic signals (Keegan and O'Mahony, 2003; Lipovac, et al., 2012).

In this study, we proposed the concept of HFI to communicate information from building facilities to users about the coordination between service robots and those facilities. We constructed an HFI system that informs the passengers in an elevator hall that a service robot is boarding the elevator and evaluated it with the experiments with general participants. A statistical analysis revealed that voice-based information significantly enhanced impressions and reduced perceived waiting time of passengers. In contrast, the statistical analysis also showed that light-based information barely improved impressions and significantly increased perceived waiting time of passengers. Our findings provide useful insights for designing future HFI systems that enhances the use of service robots in buildings. However, our study has limitations, and improvements to the system are necessary. In this study, we did not consider situations where multiple passengers are riding the elevator with a robot. The number of participants were limited, and they were recruited from the specific cultural domain (Okinawa Prefecture in Japan), which may have influenced the results. Since all participants were adult, we did not investigate whether children could understand the system. We installed our system on only one floor. In post-experiment interviews, several participants mentioned that similar guidance should be provided on other floors and inside the elevator as well. The system was developed with a focus on the robot’s elevator boarding, but guidance is also needed when a robot is exiting. When a robot is on the elevator that arrives at an elevator hall, it would be helpful to inform passengers whether the robot will exit the elevator and, if so, which direction it will move after exiting. In addition, it is essential to verify the effects of those extensions on passengers’ impressions. To support multilingual users, it may also be helpful to design background music for when a robot boards or exits the elevator, in addition to the short notification sounds already implemented. We would like to address these issues in future research and improve our system.

## Data Availability

The raw data supporting the conclusions of this article will be made available by the authors, without undue reservation.
